# Epigenetics, flavor, and tomatoes: A love triangle based on a *stable* relationship

**DOI:** 10.1093/plphys/kiaf305

**Published:** 2025-07-08

**Authors:** María Flores-Tornero

**Affiliations:** Assistant Features Editor, Plant Physiology, American Society of Plant Biologists; Departament de Biologia Vegetal, Facultat de Ciències Biològiques, Universitat de València, 46100 Burjassot, Valencia, Spain

Many people love tomatoes (*Solanum lycopersicum*) and buy them according to their appeal in terms of size, color, or, most importantly, flavor. For this reason, many studies have explored the regulation of quality traits during fruit ripening (Klee and Giovannoni 2011; Shi et al. 2022). However, flavor regulation studies lagged behind; only genes that encode biosynthetic enzymes for a few related volatiles have been characterized (Klee and Tieman 2018). In recent years, some work highlighted the relevance of RNA epigenetic changes during tomato growth and ripening, specifically N^6^-methyladenosine (m^6^A) modifications (Zhou et al. 2019; Hu et al. 2022). This epigenetic mark is recognized by proteins containing a YTH domain, playing a crucial role in stabilizing or sequestering target mRNAs (Wang et al. 2014). So far, 9 YTH-containing genes have been described in tomato (*SlYTH1-9*) (Yin et al. 2021), but only 1 has been characterized (*SlYTH2*) and identified as a negative regulator of volatile production (Bian et al. 2024). A recent study characterized *SlYTH1* by knocking out this gene and observed a plethora of phenotypic deffects, among them short roots in the seedlings, delayed vegetative growth, or reduced fruit weigth and locule number (Yin et al. 2022), but its relation with fruit ripening and flavor regulation was not addressed. In this issue of *Plant Physiology*, Gao and colleagues discovered that SlYTH1 positively regulates tomato flavor by stabilizing transcripts of enzymes involved in the synthesis of 2-isobutylthiazol (IBT) volatile, a well-known enhancer for tomator flavor.

As a first step, authors confirmed the high expression of *SlYTH1* during fruit ripening by validating RNAseq data in 3 different cultivars of tomato (Figure). Then, they used CRISPR/Cas9 to create *slyth1* knockout lines to measure by gas chromatography-mass spectrometry levels of specific compounds known to contribute to tomato flavor. They observed a consistent and significant reduction of IBT in *slyth1* compared to wild type (WT). This observation established the link between SlYTH1 and a positive regulation of IBT biosynthesis.

**Figure.**
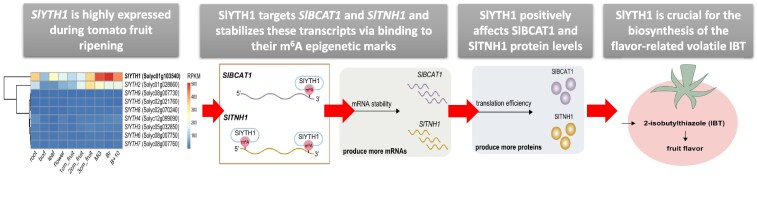
Summary diagram of the mechanism linking an RNA epigenetic modification with tomato flavor. The m^6^A reader SlYTH1 recognizes and binds to the m^6^A-modified sites on the *SlBCAT1* and *SlTNH1* transcripts. This binding stabilizes the transcripts and promotes translation efficiency, increasing the amounts of SlBCAT1 and SlTNH1 proteins and eventually triggering the production of the flavor-related volatile IBT. Figures were taken and adapted from Gao et al. (2025).

Given that IBT is synthesized by the enzymes SlBCAT1 and SlTNH1 using leucine as the precursor, the authors determined the levels of leucine and cysteine in *slyth1* tomatoes and discarded precursor absence as the cause of the reduced IBT levels. Next, they measured transcript levels of *SlBCAT1* and *SlTNH1* in both *slyth1* and WT tomatoes by quantitative PCR and observed a considerable reduction in *slyth1*. This suggested that SlYTH1 affects the stability of *SlBCAT1* and *SlTNH1* transcripts. To determine the stabilizing role of SlYTH1 in those transcripts, Gao and colleagues monitored the degradation rate of *SlBCAT1* and *SlTNH1* transcripts in *slyth1* and WT tomatoes after blocking transcription with actinomycin D. The result was a significantly faster degradation of *SlBCAT1* and *SlTNH1* transcripts in *slyth1* than in WT. This observation clearly supported the role of SlYTH1 in stabilizing *SlBCAT1* and *SlTNH1* transcripts, eventually affecting IBT synthesis in the ripening process (Figure).

However, whether SlYTH1 also influences translation efficiency needed to be answered. To this end, Gao and colleagues determined that SlYTH1 localizes in the cytoplasmic surface of the ER by coexpressing SlYTH1-GFP fusion with an ER marker-mCherry in tobacco leaves. Then, they used polysomes (clusters of several ribosomes that are translating mRNA) to measure the abundance ratio of *SlBCAT1* and *SlTNH1* transcripts compared to total RNA. The result was a significant reduction in the translation efficiency of *SlBCAT1* and *SlTNH1* transcripts in *slyth1* tomatoes, clearly showing that SlYTH1 not only stabilizes but also facilitates transcript translation of their target genes by promoting association with polysomes.

To complete the puzzle, the authors also investigated the link between m^6^A epigenetic modification recognition by SlYTH1 and the postranscriptional regulation of *SlBCAT1* and *SlTNH1* transcripts. Gao and colleagues identified high levels of m^6^A marks in those transcripts and confirmed in vivo interaction of *SlBCAT1* and *SlTNH1* transcripts with a flag-tagged SlYTH1 using RNA immunoprecipitation quantitative PCR. Finally, to prove that SlYTH1 is indeed interacting with *SlBCAT1* and *SlTNH1* transcripts specifically via m^6^A binding sites, the authors performed an RNA electrophoretic mobility shift assay assay using probes with m^6^A and A as control. A strong binding signal in probes with the m^6^A mark and its absence in the control clearly proved this protein-mRNA interaction linked to m^6^A (Figure).

These results highlight the potential of SlYTH1 manipulation to improve flavor quality, but some interesting questions remain to be explored, like the effect(s) of an overexpression of SlYTH1 in tomato flavor and in the phenotypic defects described in *slyth1*, or how conserved this mechanism is among plants.

All thogether, the interesting study from Gao et al. 2025 provides a better understanding of how flavor is regulated by the interplay between an RNA epigenetic modification m^6^A, the stabilizing role of its reader SlYTH1, and the enhanced translation of the biosynthetic enzymes that produce IBT.

## Data Availability

The data underlying this article are available in the highlighted article and in its online supplementary material.
